# General practice management after transition events: protocol for an experience-based co-design study

**DOI:** 10.3399/BJGPO.2023.0244

**Published:** 2024-09-18

**Authors:** Zakia Shariff, Jeremy Dale, Rachel Ann Spencer

**Affiliations:** 1 Warwick Applied Health, Warwick Medical School, University of Warwick, Coventry, UK

**Keywords:** general practice, transition, experience, decision making, communication

## Abstract

**Background:**

Discharge from hospital is a critical part of the patient journey, particularly for older patients with multimorbidity and polypharmacy. General practice has a key role in managing the post-discharge course of patients. A communication intervention for use in general practice in the immediate post-discharge period has great potential to improve shared decision making, enhancing patient experiences of post-discharge care.

**Aim:**

General Practice Management After Transition Events (GP-MATE) aims to produce a tool for older patients and their carers (GP-MATE), which will assist better communication with their general practice about their care after discharge, thereby improving patient safety outcomes.

**Design & setting:**

Experience-based co-design (EBCD) study involving general practices across the West Midlands.

**Method:**

A slightly modified approach to EBCD will be followed to create GP-MATE. A focused ethnography undertaken at general practices will provide an understanding of practices’ systems for post-discharge management of older patients. Semi-structured video interviews with recently discharged older patients or their carers will be edited into a trigger film. Finally, co-design workshops with older people, carers, and healthcare staff working in general practices will take place with participants from three regions across England.

**Conclusion:**

EBCD will be used to take a patient-centric approach towards creating GP-MATE; patients’ and carers’ priorities will be directly reflected within the tool. GP-MATE will be a low-cost intervention that improves health literacy, empowering patients to fill the emerging gap in continuity in the post-discharge period and enhancing patient experiences of post-discharge care.

## How this fits in

General practice has an important role in managing the post-discharge course of patients. However, there are currently very few tools to support post-discharge contact, nor any accepted structure for what consultations with older recently discharged patients should look like. GP-MATE will be a communication intervention for use in general practice in the immediate post-discharge period that aims to help improve shared decision making and enhance patient experiences of post-discharge care.

## Introduction

Discharge from hospital is a critical part of the patient journey, particularly for older patients with multimorbidity and polypharmacy. As the population of older patients increases, pressure rises in all areas of the NHS,^1^ including an enormous burden of complex care for primary care when patients return home. Data collected by the National Reporting and Learning System on discharge-related harms, including death, found that three-quarters of patients experiencing serious harm owing to incomplete discharge planning were older.^2^ There are many plausible routes to reducing these errors and harms including the design of safer systems, protected time and workspace, and better integration between primary and secondary care,^3^ but one of the least researched avenues is communicating with patients to close the error loop.^4^

A communication intervention for the immediate post-discharge period has great potential to improve shared decision making, enhancing patient experiences of post-discharge care. Primary care is uniquely placed to manage the post-discharge course of patients; it is the comprehensive health record holder, the first point of contact for patients, and the provider of ongoing care. GPs offer a unique service for their older patients in terms of medical overview of care and advocacy based on continuity from a developed relationship.^5^ However, a recent scoping review to identify existing tools for post-discharge communication found no ready-made tools specifically created for primary care.^6^ General Practice Management After Transition Events (GP-MATE) will aim to address this gap by producing a tool for older patients and their carers (GP-MATE), which will assist better communication with their general practice about their care after discharge, thereby improving patient safety outcomes.

GP-MATE will use experience-based co-design (EBCD) to create this tool, maximising patient involvement at all stages. EBCD consists of two core elements, an experience element and a participatory, co-design element. The experience element places a focus on improving the whole experience of a product or service and makes use of practical tools and concepts such as emotional mapping.^7^ The other central thread underpinning EBCD is the co-design process, where users are directly involved in the design and development of an intervention to improve a product or service.^8^

## Method

**Figure 1.**
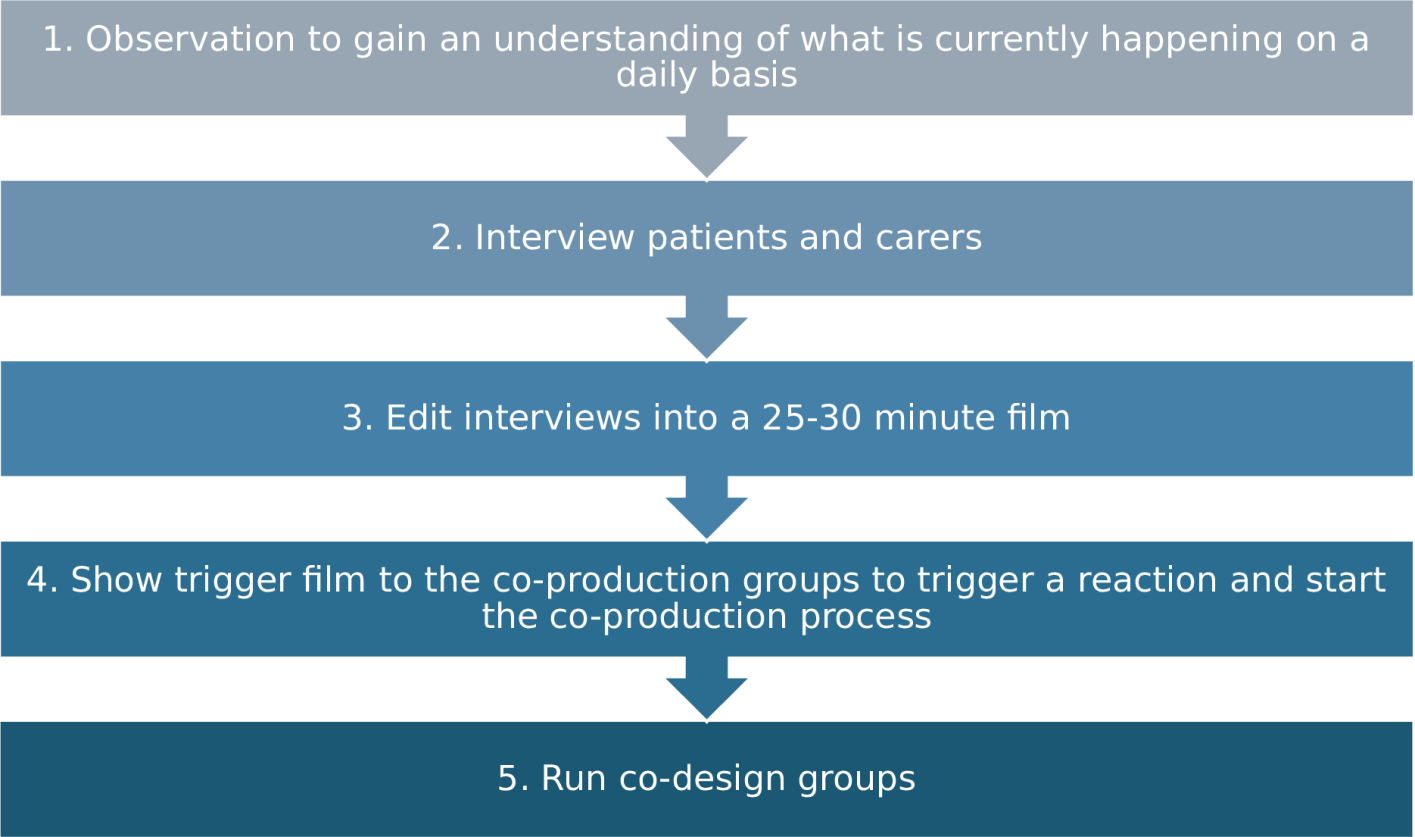
Stages of the evidence-based co-design process as modified for GP-MATE

GP-MATE uses a slightly modified approach to EBCD. While generally, EBCD is conducted within a single secondary care organisation as part of a quality improvement process,^9^ there is growing evidence of its potential to be used within larger intervention development studies.^10^ GP-MATE uses EBCD as part of a larger study, in which the tool developed is taken forward in a feasibility study to test acceptability and usability. The overall aim is to provide evidence such that the National Institute for Health and Care Excellence (NICE) can recommend GP-MATE to be adopted into routine practice. Patients are therefore being recruited from a range of practices, resulting in a wider demographic of patients and carers and a reflection of multiple organisations’ systems. Furthermore, while EBCD is generally conducted using the same participants for both the experience and co-design elements, we are recruiting different cohorts of patients to ensure a diverse group of participants representative of the heterogeneous older population. This slightly modified approach is illustrated in Figure 1 and is similar to the approach described by Bate and Robert.^11^

### Observation to gain an understanding of what is currently happening

EBCD starts by using ethnographic methods to gain an insight into how a service works and what patient and staff experiences may be.^12^ To recruit practices for this stage, the study team will work alongside the West Midlands Clinical Research Network (WM CRN). Purposeful sampling will be used to select 10 practices based on size, geography (rural or urban), ethnic diversity, and socioeconomic status. Older patients that have been discharged from hospital in the past 3 months or their carers will be recruited from the 10 practices.

Systems information will be gathered from fieldwork at practices, including informal discussions with administrative and clinical staff. Interview guides have been developed informed by previous research, which found that completing requests for follow-up tests, implementing medication changes, and arranging follow-up appointments were areas where failures in processing requested actions were identified.^13^ Interview guides consider these key areas, while also considering the discharge process as a whole. The insights gained will be used to create a process map of actions taking place in primary care once a patient leaves hospital.

A number of methods are available to illustrate these process maps. GP-MATE will use the Functional Resonance Analysis Method (FRAM),^14^ a technique that has been used successfully in primary care. FRAM has previously been used to identify key sources of variability in a system and the remedial actions that are needed to help minimise disruption caused by this variability.^15^

### Interview patients and carers and edit interviews into a 25–30-minute film

The sample of patients and carers recruited from stage one will first be screened by the lead GP at each practice so that patients for whom the practice does not hold carer details and who have severe mental health problems or cognitive impairment will be excluded. Eligible patients will be invited to take part in a filmed interview that records important experiences and emotions of, as well as insights into, the person’s journey.^16^ We aim to interview 10 older patients or their informal carers. This is to maintain the quality of the film; >10 participants can result in viewers finding the narrative difficult to follow.^12^ The film will be shown to staff and patients to impactfully convey how patients currently experience post-discharge care.^12^ The film becomes an important catalyst for improvement; seeing and listening to patients allows staff to connect to patients and provides a persuasive starting point for implementing change.^17^

Participants will be interviewed on film in their own homes either face-to-face or via video link. The interview guide has been developed using a journey format,^18^ allowing participants to talk about their post-discharge experiences and the communication that followed with their GP practice with minimal interruption. Their needs and preferences for the content and format of GP-MATE will also be explored. A key outcome of these interviews is the identification of ‘touchpoints’, defined as both positive and negative interactions between staff and patients that are perceived as being crucial to the overall experience of care.^19^

Each individual film will be edited down to provide a 30-minute 'trigger film'.^12^ This process can be labour intensive and there is a need to avoid any potential for bias; quotes from every participant and both positive and negative experiences will be included.^12^ Patients themselves will have the final say on the clips included from their individual interview.^12^

When considering this part of the EBCD process, there are a number of ethical considerations to take into account. The techniques used to address these in GP-MATE are summarised in Table 1.

**Table 1.** Methods for addressing ethical issues during the creation of the trigger film for GP-MATE

Ethical consideration	Method used to address consideration in GP-MATE
Ethical issues arising from interviewing a vulnerable population group	Patients will be interviewed by an academic GP and/or a community pharmacist who is used to speaking to frail and vulnerable older people after discharge from hospital.
Potential for safety of care issues being raised during the video interview	All issues raised will be handled sensitively and the researcher and/or clinician interviewer will understand if a safety of care issue has been raised that needs reporting back to the patients’ care team. The need for a researcher to disclose any evidence of serious professional misconduct or safeguarding concern will be made clear in the relevant participant information sheets.
Potential for patient to become distressed during the video interview	Interviews can be stopped at any time should the participant wish to do so or becomes distressed.
Potential for identification of patients who lack capacity to consent to appear on video	When a patient lacks capacity to consent to appear on film, personal consultee agreement from their next of kin will be sought and/or their carer interviewed instead.
Need for consent for the video to appear online in the public domain	The film will be available publicly in perpetuity and this will be explicit in the consent forms produced.

### Show trigger film to the co-production groups

Presentation of the film to the co-production groups will kick-start the co-production process. During the film, participants will write down any thoughts or reflections, after which an emotional mapping exercise will be undertaken. Emotional mapping will provide patients and staff the opportunity to describe in detail both the positive and negative emotions experienced along the patient journey.^16^ Following identification of all touchpoints from watching the trigger film, participants will be asked to select key emotions associated with each touchpoint. Touchpoints associated with mainly negative emotions will be prioritised for further action.^16^

Participants will also be introduced to simplified FRAM diagrams (generated in stage one) of the processes occurring in general practice to help them understand the parts of their post-discharge journey they do not see (the healthcare administrative side). With facilitation from the research team, co-design groups will attempt to overlay the process maps with the results of the emotional mapping from the film, melding the patient and staff experience together for the first time.

### Run co-design groups

The co-design process is intended to be a participatory approach to developing an intervention that brings together staff, patients, and carers to design local solutions to local problems.^20^ The co-design groups in GP-MATE will create a prototype of the communication tool (GP-MATE). The tool itself will be made up of the following three components: 1) a communication tool for patients, carers, and general practice staff; 2) a learning set for general practice staff that includes the implementation strategy for the communication tool; and 3) a space for the interaction between patient or carer and healthcare professional in the general practice appointment book.

The co-design groups will consist of patients (aged ≥65 years) and their informal carers and general practice staff in three different areas of England. Three half-day workshops will be organised (5–8 older people and/or carers per regional group) and data will be analysed iteratively so that emerging results feed into future meetings. The first workshop (as described in stage four) will use the trigger film to simulate discussion on what is important post-discharge. The second meeting will focus on the content of GP-MATE and how it will bring about change. The final meeting, consisting of patients and carers, will involve reviewing previous findings, including findings in relation to the separate staff focus group conducted, and deciding the final intervention.

The staff focus group that feeds into the final patient and carer workshop will be conducted with general practice staff in each of the three regions. A half-day workshop will be conducted where staff will watch the trigger film and will discuss how the intervention fits with available resources. Staff will be asked for ideas on content, format, and usability of GP-MATE and their requirements for a learning set for professionals to assist implementation.

The mixed co-production group will aim to reach consensus on the content of the final prototype tool. Six patient and/or carer representatives and three practice staff members will meet with the research team at this combined event.

## Discussion

### Summary

EBCD will be used within GP-MATE to develop a tool that will help improve patient communication with general practice following discharge. Patients’ and carers’ priorities will be directly reflected within the tool, which will then be tested in a feasibility study in a general practice setting.

### Strengths and limitations

One of the key strengths of the EBCD approach is the flexibility it offers. Primary care organisation is different to secondary care and research methods often need to be adapted to fit. Service development is also different in the primary care setting; it is rare that individual general practices could afford the time and effort required to be invested in a method such as EBCD. Adapting the approach by recruiting a number of general practices helps to overcome this issue while also allowing for the study of multiple organisations’ systems.

Other strengths of this approach include the opportunity to engage and empower patients and staff; EBCD ensures that the patient experience is heard and that an implementation framework is developed that is context sensitive. Staff especially enjoy using EBCD; staff who listen to patient experiences on film are often highly impacted, with previous participants discussing the *'light-bulb moment'* that can arise when listening to personal experiences.^17^

There are some limitations to using EBCD, the main one of which is the timescale and associated costs. An accelerated approach, where individual patient videos are replaced with existing videos from an archive,^12^ can be used to overcome this limitation. Other limitations include the potential for conflict and tension to arise between patients and staff that often relate to issues of power.^21^ The research team need to be aware of this and ensure that co-design groups are run appropriately.

### Implications for practice

GP-MATE will be a low-cost intervention that improves health literacy, empowering patients to fill the emerging gap in continuity post-discharge. Very few tools currently exist that support primary care staff in post-discharge contact, nor is there any accepted structure for what consultations with frail, older, and recently discharged patients should look like. This research will address these healthcare professional needs directly. In using GP-MATE to improve patient health literacy, secondary care and patients may also benefit from reduced readmissions.
